# Labor market exit around retirement age in Sweden and trajectories of psychotropic drugs in a context of downsizing

**DOI:** 10.1186/s12889-020-08589-6

**Published:** 2020-05-05

**Authors:** Sandra Blomqvist, Hugo Westerlund, Kristina Alexanderson, Linda L. Magnusson Hanson

**Affiliations:** 1 Stress Research Institute, Stockholm University, SE-106 91 Stockholm, Sweden; 2 Division of Insurance Medicine, Department of Clinical Neuroscience, Karolinska Institutet, SE-171 77 Stockholm, Sweden

**Keywords:** Downsizing, Psychotropic drugs, Sick-leave, Disability pension, Unemployment, Retirement, Longitudinal analysis

## Abstract

**Background:**

A maintained psychological wellbeing is important in order to continue working longer and remain active into older age. However, little is known about impact of different organizational factors, such as downsizing, on the mental health of older workers exiting the labor market. The aim in this study was to investigate trajectories of purchases of psychotropic drugs in relation to labor market exit later in life in a context with and without downsizing.

**Method:**

People living in Sweden, born 1941–1951, exiting paid work via unemployment, sickness absence/disability pension, or old-age pension were followed from 2005 to 2013 regarding purchases of psychotropic drugs. Individuals employed at a workplace closing down or downsizing with ≥18% between two subsequent years were compared to employees exiting from workplaces without downsizing or workplace closure. Generalized estimating equations was applied to derive trajectories of annual prevalence of purchased antidepressants, sedatives and anxiolytics from 4 years before to 4 years after a labour market exit.

**Results:**

During the period around the exit, old-age retirees experiencing a downsizing/workplace closure did not decrease their purchases of sedatives (OR 1.01 95% CI 0.95–1.07) while the unexposed decreased their purchases during this period (OR 0.95 95% CI 0.92–0.98). Similar differences concerning sedatives and antidepressants between exposed and unexposed were seen for those exiting via sickness absence or disability pension. Furthermore, a significant difference in purchases of anxiolytics was observed between those exposed to downsizing (OR 1.10 95% CI 0.97–1.24) and the unexposed (OR 0.98 95% CI 0.91–1.06) exiting via old-age retirement during the time before the exit.

**Conclusion:**

Downsizing or workplace closure, although weakly, was associated with higher prevalence of psychotropic drugs certain years around the labor market exit. The results support the idea that involuntary labor market exit in mature adulthood may negatively affect the development of mental health.

## Background

Across most of the industrialized world, people are living longer and also continue to working longer into early old age. An important challenge is therefore to promote healthy aging allowing people to work longer and remain active and independent in later life. It has been emphasized that psychological wellbeing of older individuals is important for healthy aging [1]. However, a greater understanding of how work life and transitions out of employment may affect the mental health of older people is needed.

We know from previous studies that poor working conditions are associated with poor mental health [2, 3]. Also exiting an employment can be associated with poor mental health [4]. Unemployment has shown detrimental effects on mental health [5, 6]. Less research has, however, addressed possible determinants of the development of mental health across the transition to retirement. Research on the association between retirement and mental health has shown inconclusive findings. One review concluded that there is strong evidence for beneficial effects of retirement on mental health [7]. However, several longitudinal studies published after the aforementioned review have shown less support for beneficial effects on retirement on mental health [8–10]. These inconsistent findings may be explained by a variation across different subgroups.

When it comes to retirement a number of factors, divided into push and pull factors, influencing the decision to retire have been investigated in the previous literature. Pull factors such as economic incentives, a retired spouse and being able to spend more time on social life are factors that can make retirement an attractive option [11], while ill health, organizational factors and poor working conditions might push people into retirement [11, 12] These factors act side by side but a predominance of push factors could mean that the retirement is perceived as involuntary [11, 13]. Studies examining different exit routes have suggested that exit due to poor health, via disability pension, is associated with beneficial effects on mental health, but that the post-retirement mental health is still worse than that of old-age retirees [9, 10, 14, 15]. Also, the impact of different push and pull factors on exiting voluntarily or involuntarily from the labor market has been studied, suggesting that high work pressure, low control, low job satisfaction, and poor health is associated with involuntary exit, either via disability pension, unemployment, or early retirement [11, 12, 16]. One study found a dose-response association, with more push factors associated with more negative effects on psychosocial wellbeing after retirement [17]. However, few studies have investigated all three aspects; push factors, exit routes and health after the exit. Furthermore, to our knowledge, only one study [18], using a sample from a single company, has looked closer into downsizings, exit and mental health after the exit. That study compared levels of psychological distress among employees staying in a workplace after a downsizing with that of employees who retired voluntary or involuntary. It was found that those who retired involuntary reported the highest levels of psychological distress while voluntary retirees reported the lowest levels [18].

Organizational downsizing, is a common feature in the working life today, and there are indications that downsizings are associated with poorer mental health, although the evidence of an association is still insufficient largely due to heterogeneity in measurements of mental health and use of study designs, limiting the possibility to draw conclusions about causality [19]. Some previous studies have indicated that being exposed to downsizing affects mental health negatively measured in terms of increasing purchases of psychotropic drugs [20–22]. Anticipating an organizational change has been suggested to be as negative as an actual event in terms of self-reported levels of anxiety and depressive symptoms [23]. However, others have not found an effect during the anticipation phase and only transitory effects on mental health among those who were actually laid off [24].

In this study we aimed to increase the knowledge about the association between downsizing, as a possible push factor for labor market exit, and mental health around labor market exit, by investigating trajectories of purchases of psychotropic drugs both before and after labor market exit by different routes from a workplace with and without a preceding downsizing.

## Methods

### Study population

**Fig. 1:**
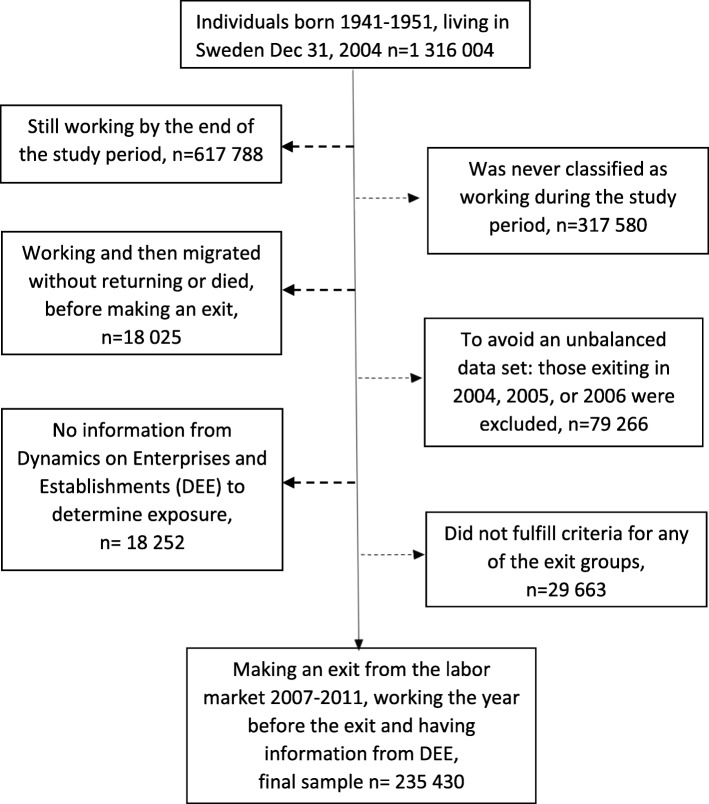
Flow chart describing the inclusion/exclusion criteria of the analytical sample

In this prospective cohort study, data from the Insurance Medicine All Sweden (IMAS) database were used, linking micro-level information from the Longitudinal Integration Database for Health Insurance and Labor Market Studies (LISA) and Statistics on Dynamics of Enterprises and Establishments (DEE) held by Statistics Sweden and the Swedish National Prescribed Drug Register held by the Swedish Board of Health and Welfare, by Swedish personal identity numbers. We used information on all individuals born 1941–1951 and living in Sweden on 31 December 2004 (*n* = 1,316,004), then aged 53–63 years. These individuals were followed with regard to purchases of psychotropic drugs from 4 years before to 4 years after a labor market exit, thus people still working at the end of the study period, 2012–2013, were excluded, (*n* = 617,788) as well as those never classified as working during this period (*n* = 317,580). People who were in paid work the year before they died or emigrated without returning during the study period were also excluded (*n* = 18,025). To get an analytical sample with a balance of information on drug purchases before and after a labor market exit, individuals exiting between 2004 and 2006 were excluded (*n* = 79,266) since information on purchases of psychotropic drugs is only available from 2005. For people exiting earlier, only limited information about purchases were thus available. Furthermore, *n* = 18,252 were excluded because information on workplace from the DEE statistics was missing completely or in the year before the individual exited. Lastly, 29,663 individuals were excluded since they did not fulfill the unemployment, sickness absence/disability pension, or old-age pension criteria described below. This resulted in an analytic sample of 235,430 women and men, see Fig. 1 for a graphical overview.

### Labor market exits

In order to identify an exit, we categorized people annually as either being 1) gainfully employed (including self-employed), (classification made by Statistics Sweden based on annual income from work exceeding some minimal amount determined and calibrated by sociodemographic factors by Statistics Sweden in order to correspond with other statistics [25, 26]) (2) unemployed (received unemployment benefits during > 180 days that year), 3) on sickness absence/disability pension (SA/DP) (having received SA benefits for > 180 days or disability pension > 1 month), 4) old-age retirement (receiving any old-age pension that year + an old-age pension of at least 90,000 SEK the following year and that exceeding income from work). In order for a change to be defined as an exit, it should have been preceded by at least 1 year of gainful employment. In addition, an individual defined as exiting had to fulfill the criteria for belonging to group 2, 3, or 4 for the rest of the study period (a minimum was the two final years) since permanent rather than temporary exits was of interest. The studied exits occurred from 2007 to 2011.

### Downsizing

In order to relate purchases of psychotropic drugs to downsizings/workplace closure we used information from the DEE register on structural changes within establishments and organizations. Via information on main employer in the LISA register it was possible to obtain workplace data from the DEE register. Employees at establishment that reduced their staffing by ≥18% between two consecutive years, from 1 November 2006 to 31 October 2011, and where the latter year coincided with their exiting year, were classified as exposed to an organizational downsizing. The cut-off ≥18% has been used in previous studies on major downsizing [22, 27, 28]. We also included employees experiencing workplace closure, which would be the most severe type of downsizing where everyone is laid off. This resulted in 70,038 exposed employees, almost a third of the total sample. Further on, the term downsizing will be used, including also those exposed to workplace closure.

### Purchases of prescribed psychotropic drugs

In Sweden, psychotropic drugs can only be purchased at a pharmacy with a prescription. Information on drugs dispensed at pharmacies was retrieved from the Swedish National Prescribed Drug Register between November 2005 and November 2013. Prescriptions coded N06A (antidepressants), N05B (anxiolytics), and N05C (hypnotics and sedatives) according to the Anatomical Therapeutic Chemical (ATC) classification system were extracted from the register with regard to the date of purchase. We created three dichotomous variables indicating whether the individual had bought any antidepressants, anxiolytics, or sedatives, respectively, during each 12-month period (matching the 12 month periods used to define downsizing) from 4 years before, during, and 4 years after the exit.

### Covariates

The following sociodemographic factors, obtained from LISA, were assessed in the beginning of the 12 month period of exit; sex, age (continuous), income from work (continuous), educational level (‘primary education’, ‘secondary education; high school’, ‘post-secondary education (college/university) <3 years’ and ‘post-secondary education ≥3 years.’), family status (‘living with partner’, ‘not living with partner’, (that is, married or cohabitant) (both irrespective of children living at home)), type of living area (‘big city’ (i.e., metropolitan areas with more than 90,000 inhabitants), ‘medium-sized city’(i.e., areas with 27,000 to 90,000 inhabitants within a 30 km radius from the largest municipality center, ‘small city/village’ (i.e., areas with < 27,000 inhabitants within a 30 km radius from the largest municipality center)). All these factors were assumed to be associated with exit and/or downsizing and psychotropic drug purchase and hence suitable to adjust for in the analysis, according to a Directed Acyclic Graph (available from the main author).

### Data analysis

To investigate the prevalence of purchases of prescribed psychotropic drugs in relation to labor market exit we used Generalized Estimating Equations (GEE) by means of the SAS software. Since we were interested in the time before and after the exit, rather than certain 12-month periods, we centered all observation around *t* 0 (period of exit) and created a relative time variable indicating 12-month period in relation to exit, ranging − 4 to + 4. Thus, the trajectories were based on nine potential observations per individual. Since prescription data were available only between 2005 and 2013 some individuals had missing data at time − 4, − 3, and + 4. However, the working correlation structure was estimated using the all available pairs method allowing for intermittent missing and dropout.

Different correlation structures were compared with regard to QIC-values (Quasi-likelihood under the independence model criterion) [29]. Since one of the strengths of using GEE is the possibility to account for intra-individual correlation, we chose to apply the autoregressive correlation structure, displaying the second lowest QIC value, rather than the independent correlation structure which displayed a slightly lower QIC value.

To test for time trends, we defined three time periods in connection with labor market exit; ‘pre-exit’ (from 12-month period − 4 to − 1), ‘peri-exit’ (− 1 to + 1) and ‘post-exit’ (+ 1 to + 4). Odds ratio (OR) and 95% confidence intervals (CI) of purchasing antidepressants, sedatives, and anxiolytics, respectively, were calculated comparing the prevalence of purchases in the first and the last 12-month period in each of the three time periods. In the peri exit period for example, the estimates ORs thus contrasted the odds of purchase at time + 1 with that of time − 1. First, we only adjusted for calendar time (see Additional file 1). Secondly, we included sociodemographic factors (presented in the Result section). Differences in purchase patterns were further tested between employees exposed and unexposed to organizational change before their exit, within each exit group, respectively, by contrasting the odds ratios between exposed and unexposed during pre-exit, peri-exit and post-exit and calculating 95% confidence intervals.

## Results

A description of the sample is provided in Table 1. In total, 235,430 people exited paid work during the studied years. The majority exited via old-age retirement (*n* = 166,273), followed by sickness absence or disability pension (SA/DP) (*n* = 45,463), and unemployment (*n* = 23,694). A larger proportion of the women were found among those exiting via SA/DP while there was a larger proportion of the men among those exiting via unemployment.

**Table 1:** Descriptive statistics by type of exit and whether exposed/not exposed to downsizing or workplace closure

	Unemployed		Sickness absent/disability pension		Old-age retirement	
	Downsizing	No Downsizing	Downsizing	No Downsizing	Downsizing	No Downsizing
	n (%)	n (%)	n (%)	n (%)	n (%)	n (%)
	8599	15,095	14,620	30,843	46,819	119,454
Sociodemo-graphic Information						
Mean age (sd)	60.4 (2.2)	60.2 (2.3)	61.5 (2.4)	61.7 (2.3)	63.9 (2.2)	63.7 (2.2)
Men	5085 (59.1)	8431 (55.9)	6996 (47.9)	10,160 (32.9)	27,229 (58.2)	55,461 (46.4)
Women	3514 (40.9)	6664 (44.1)	7624 (52.3)	20,683 (67.1)	19,590 (41.8)	63,993 (53.6)
Education						
Primary	2970 (34.6)	4450 (29.5)	4821 (33.0)	8635 (28.1)	13,475 (28.8)	30,664 (25.7)
Secondary	4260 (49.6)	7507 (49.8)	6828 (46.8)	14,028 (45.5)	20,952 (44.8)	52,441 (43.9)
Post-secondary <3 years	389 (4.5)	721 (4.8)	392 (2.7)	598 (1.9)	1680 (3.6)	3479 (2.9)
Post-secondary ≥3 years	974 (11.3)	2407 (15.9)	2549 (17.5)	7547 (24.5)	10,651 (22.8)	32,764 (27.5)
Mean Income (in 100 SEK) (sd)	2243 (1944)	2312 (2734)	2270 (7024)	2060 (1862)	2933 (5032)	2612 (3974)
Living with partner	5306 (61.7)	9216 (61.1)	9157 (62.6)	19,371 (62.8)	32,454 (69.3)	81,582 (68.3)
Not living partner	3293 (38.3)	5879 (38.9)	5463 (37.4)	11,472 (37.2)	14,365 (30.7)	37,872 (31.7)
Type of living area						
Big city	2408 (28.0)	4287 (28.4)	4072 (27.8)	9091 (29.5)	16,460 (35.2)	41,235 (34.5)
Medium-sized city	3233 (37.6)	5711 (37.8)	4865 (33.3)	11,528 (37.4)	15,989 (34.1)	43,388 (36.3)
Small city/village	2958 (34.4)	5097 (33.8)	5683 (38.9)	10,224 (33.1)	14,370 (30.7)	34,831 (29.2)

Legend: Information on sociodemographic factors, from the beginning of the year of among people born 1941–1951 who had a labor market exit in Sweden in 2007–2011 (*n* = 235,430)

Exit via old-age retirement was equally common among women and men. In total, nearly 30% (*n* = 70,038) were exposed to downsizing in connection to their exit. Comparing the three exit groups, a larger proportion of the old-age retirees had higher educational level, lived with a partner, and in a big city, and were somewhat older compared to the SA/DP group and the unemployed. When comparing those exposed to downsizing to those unexposed some differences emerged. In general, the exposed had lower educational level and a higher proportion of the men than the women seemed to experience a downsizing event before exiting the labor market. Otherwise, with regard to age, cohabitation, and type of living area, the exposed and unexposed were similar within each exit group.

### Purchases of psychotropic drugs before and after exiting the labor market

Trajectories of purchases of antidepressants, sedatives and anxiolytics were fitted for the three groups; SA/DP, old-age retirement, and unemployment, respectively. For each type of psychotropic drug and exit, trajectories were compared between employees exposed to downsizing in connection to their exit and employees making the same type of exit but without such an event. Regardless of the type of drug, employees exiting through SA/DP were the group with highest prevalence of purchasing psychotropic drugs, followed by the unemployed, while old-age retirees had the lowest prevalence. Comparing employees making the same type of exit across the three types of drugs, purchases of antidepressants and sedatives were more common than purchases of anxiolytics.

### Making an exit via old-age retirement, with or without a preceding downsizing

**Fig. 2:**
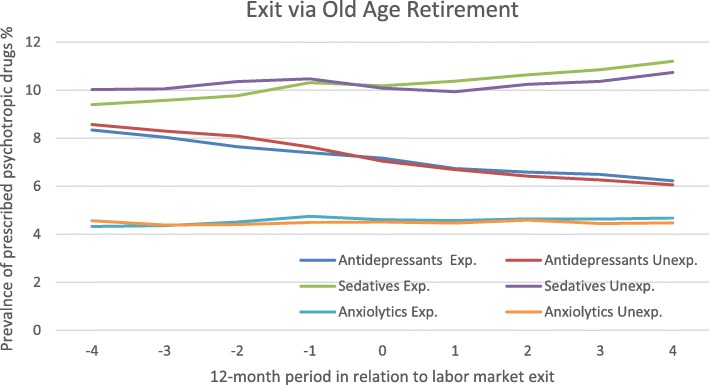
Prescribed psychotropic drugs among people exiting via old-age retirement, exposed or unexposed to downsizing Legend: Estimated prevalence (%) with 95% CI, adjustments was made for sociodemographic factors. Unexp. = Unexposed, Exp. = Exposed

For those who exited with old-age pension, exposed and unexposed, trajectories of purchases of prescribed psychotropic drugs, adjusted for calendar time and sociodemographic factors are presented in Fig. 2. The results showed a slow but steady decrease in antidepressant purchases across the whole period, from about 8% to 6%. According to the OR there was a significant decrease in purchases of antidepressants after exit among those not exposed to a downsizing event in relation to their exit (Table 2), which was not seen among the exposed. However, further tests comparing the two groups displayed no significant differences in change over time between exposed and unexposed in any of the periods.

**Table 2:** Odds ratios for purchases of psychotropic drugs by exit group and type of drug

	Pre-exit period	Peri-exit period	Post-exit period
	’-1 vs − 4’	’ + 1 vs − 1’	’ + 4 vs + 1’
Old-age retirement			
*Antidepressants*			
Downsizing	0.89 (0.80–0.98)	0.91 (0.85–0.98)	0.92 (0.84–1.02)
No downsizing	0.89 (0.84–0.95)	0.88 (0.84–0.91)	0.91 (0.86–0.96)
*Sedatives*			
Downsizing	1.10 (1.00–1.20)	1.01 (0.95–1.07)*	1.08 (1.00–1.17)
No downsizing	1.04 (0.99–1.10)	0.95 (0.92–0.98)	1.08 (1.03–1.13)
*Anxiolytics*			
*Downsizing*	1.10 (0.97–1.24)*	0.96 (0.89–1.04)	1.02 (0.92–1.14)
*No downsizing*	0.98 (0.91–1.06)	0.99 (0.95–1.04)	1.00 (0.94–1.07)
SA/DP			
*Antidepressants*			
Downsizing	1.19 (1.07–1.32)	0.88 (0.82–0.94)**	0.78 (0.70–0.86)
No downsizing	1.22 (1.14–1.32)	0.80 (0.76–0.84)	0.80 (0.75–0.86)
*Sedatives*			
Downsizing	1.21 (1.09–1.35)	0.99 (0.93–1.06)**	1.00 (0.90–1.10)
No downsizing	1.22 (1.13–1.31)	0.89 (0.84–0.93)	0.94 (0.88–1.01)
*Anxiolytics*			
*Downsizing*	1.25 (1.09–1.44)	0.93 (0.85–1.01)	0.92 (0.82–1.03)
*No downsizing*	1.25 (1.13–1.38)	0.90 (0.85–0.96)	0.93 (0.85–1.01)
Unemployment			
*Antidepressants*			
Downsizing	0.94 (0.78–1.14)*	0.90 (0.80–1.02)	0.77 (0.65–0.92)
No downsizing	0.76 (0.67–0.87)	0.80 (0.73–0.87)	0.70 (0.62–0.79)
*Sedatives*			
Downsizing	0.92 (0.77–1.10)	0.86 (0.76–0.97)	0.86 (0.73–1.01)
No downsizing	0.91 (0.80–1.04)	0.87 (0.80–0.95)	1.00 (0.89–1.13)
*Anxiolytics*			
*Downsizing*	0.81 (0.64–1.03)	0.93 (0.79–1.09)	0.96 (0.77–1.19)
*No downsizing*	0.92 (0.78–1.10)	0.88 (0.78–0.99)	0.84 (0.72–0.98)

Legend: Odds ratios were adjusted for calendar time and sociodemographic factors from the beginning of the 12-month period of exit, including sex, age, income, education, family status, type of living area, (* *p* < 0.05, ** *p* < 0.001 indicating a statistically significant difference in time trends between exposed and unexposed) (*n* = 235,430)

Both exposed and unexposed old-age retirees had similar upturning trends of purchasing sedatives. However, in the peri-exit phase the unexposed to downsizing decreased significantly OR 0.95 (95% CI 0.92–0.98) while the exposed remained unchanged OR 1.01 (95% CI 0.95–1.07) (Table 2). The difference was significant according to the test comparing the two groups.

In general, the prevalence of purchases of anxiolytics was rather low among old-age retirees, around 5%. In the pre-exit period (− 4 to − 1) those exposed to downsizing had an OR of 1.10 (95% CI 0.97–1.24) while the unexposed had an OR of 0.98 (95% CI 0.9–1.06). There was a significant difference between the groups when comparing change in prevalence in this period.

### Making an exit via sickness absence or disability pension, with or without a preceding downsizing

**Fig. 3:**
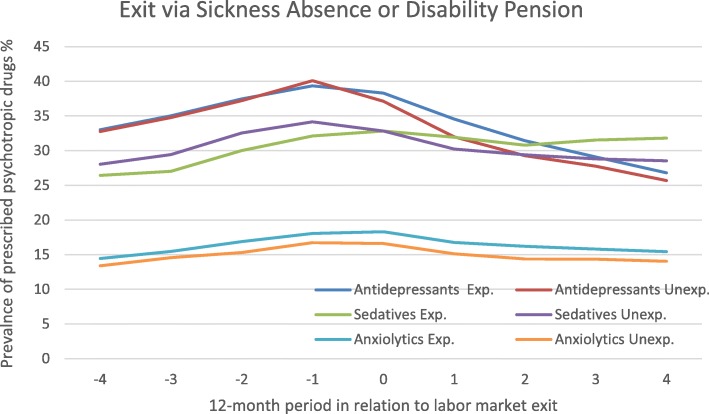
Prescribed psychotropic drugs among people exiting via sickness absence/disability pension exposed or unexposed to downsizing Legend: Estimated prevalence (%) with 95% CI, adjustments was made for sociodemographic factors. Unexp. = Unexposed, Exp. = Exposed

Figure 3 shows the trajectories of estimated 12-month prevalence of purchases of psychotropic drugs, among those exiting via SA/DP. The prevalence of antidepressant purchases increased during the pre-exit period among those exposed to downsizing as well as the unexposed, from about 33% to 40%. In the peri-exit period, the prevalence in both groups started decreasing. Although CIs overlapped when comparing the trends in prevalence during the peri-exit period (Table 2), the unexposed (OR 0.80, 95% CI 0.76–0.84) decreased more than the exposed (OR 0.88, 95% CI 0.82–0.94).

Regarding purchases of sedatives, those exposed to downsizing had an increasing prevalence from around 26% to 33% during the pre-exit period that remained stable during the peri-exit period. Thereafter, the prevalence decreased slightly but returned again to 33% by the end of the post-exit period. The unexposed SA/DP group increased their purchases of sedatives during the pre-exit period, from 28 to 34%, then decreased during the peri-exit and post-exit period, returning to the initial level of 28%. According to OR (Table 2), purchases generally decreased among the unexposed in the peri-exit period (OR 0.89, 95% CI 0.84–0.93), while they did not among the exposed (OR 0.99, 95% CI 0.93–1.10). According to the test comparing the two groups the difference was statistically significant. The steady decrease among the unexposed meant that the trajectories of unexposed and exposed in the SA/DP group crossed over at year 0, leaving the exposed with a higher prevalence in the post-exit phase compared to the unexposed.

Those exposed to downsizing in connection to their SA/DP exit had a higher prevalence of purchasing anxiolytics compared to the unexposed. Both groups showed a steady increase during the pre-exit period but the prevalence eventually started decreasing. The exposed had a prevalence below 15% in year − 4, increased to a bit more than 18% at year 0 and returned to 15% again at + 4. Corresponding figures for the unexposed was 13%, 16%, and 14%.

### Making an exit via unemployment, with or without a preceding downsizing

**Fig. 4:**
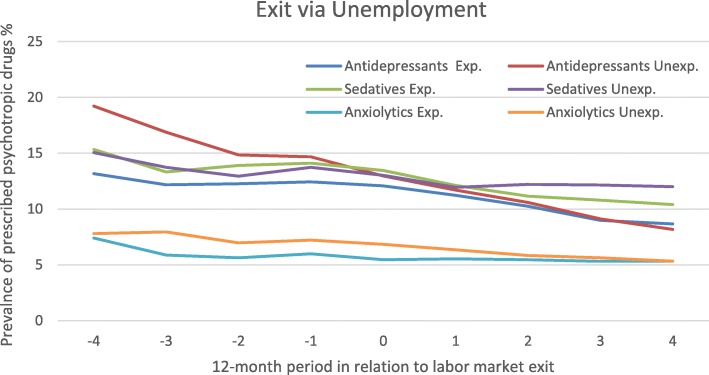
Prescribed psychotropic drugs among people exiting via unemployment, exposed or unexposed to downsizing. Legend: Estimated prevalence (%) with 95% CI, adjustments was made for sociodemographic factors. Unexp. = Unexposed, Exp. = Exposed

For the unemployment exit group, purchases of psychotropic drugs are displayed in Fig. 4. The pattern of purchasing antidepressants differed somewhat between those exposed and those unexposed to downsizing. Although the unexposed had a higher prevalence at the beginning of the period, the prevalence decreased across the whole study period, from 19% to 8%. The adjusted OR indicated a significant decrease among the unexposed in all three periods (Table 2). In the exposed group, the prevalence remained unchanged until the time of the exit and then started decreasing. When comparing those exposed to downsizing to the unexposed, there was a significant difference in purchases of antidepressant during the pre-exit phase, where exposed decreased their purchases but the unexposed did not. The overall decrease was considerably smaller among the exposed across the whole period, from 13% to 9%.

Exposed and unexposed employees becoming unemployed had decreasing and overlapping trajectories of purchased sedatives. The trend was, however, interrupted during the time before the exit, somewhat more in the exposed group. Adjusted contrasted ORs showed a significant decrease in both groups during the peri-exit phase (Table 2). The trajectory indicated a steeper decrease in the post-exit phase among the exposed compared to the unexposed. However, this difference was not confirmed in the test comparing ORs between the two groups.

When comparing the ORs of purchases of anxiolytics between exposed and unexposed exiting via unemployment, the unexposed showed a significant decrease in the peri- and post-exit phases while the exposed did not change significantly during the same periods (Table 2). The trajectories indicated an overall decrease in both groups, but whereas the unexposed decreased across the entire period, the exposed made a drop during the first year in the pre-exit period and then remained more or less on the same level throughout the rest of the study period. However, no group differences were supported in the tests comparing changes in prevalence across time.

## Discussion

In this prospective population-based cohort study we followed 235,430 employees, born between 1941 and 1951, from 4 years before they exited the labor market to 4 years after, with regard to purchases of prescribed psychotropic drugs in a context with or without an organizational downsizing. Downsizing was used as a proxy for capturing an extrinsically motivated exit, an exit that could be perceived as involuntary not motivated by individual behavior or health status. In general, the results indicated that downsizing was weakly associated with increased purchases of psychotropic drugs and negatively affecting the development of mental health during the transition to retirement.

Among people exiting via old-age retirement, those not exposed to downsizing displayed a decreasing trend in purchases of sedatives during the peri-exit period, which could not be found among the exposed. Our findings in the exposed group are in accordance with literature suggesting no beneficial effect of retirement on sleep disturbances or use of sedatives [10], while the decrease of sedative purchases around exit found in the unexposed group, is in accordance with research suggesting that retirement has beneficial effects on sleep [30]. Although the differences were only moderate, the findings could be interpreted as supportive of studies stressing the importance of voluntariness in retirement transition in order for it to have favorable or non-detrimental effects on mental health [13, 18, 31].

Furthermore, purchases of anxiolytics increased among the exposed old-age retirees before the exit while it did not among the unexposed. This is in line with previous research, finding adverse effects on mental health during the anticipation phase of the downsizing event or exit [22, 23, 32]. Uncertainty about the future and poorer working conditions during this period may act as potential mechanisms [33, 34]. Previous studies on trajectories of psychotropic drugs around retirement have mainly looked at antidepressants, showing an overall increasing trend but no change in connection to the retirement [9] or a decrease around retirement [14]. People exiting via old-age retirement in this study appeared to have an overall decrease in antidepressant purchases across time but no change around the time of the exit and the pattern did not differ by experience of downsizing or workplace closure before the exit.

Among those exiting via SA/DP, the unexposed displayed a steeper decline in purchases of antidepressant and sedatives during the time around the exit than the exposed. A decrease of purchases of psychotropic drugs after exiting with disability pension is in accordance with previous research [9, 10, 14]. Regardless of being exposed to downsizing or not, exiting via disability pension is likely to be perceived as involuntary in itself [35]. However, it is worth pointing out that others have found that objectively forced exits, such a potential downsizing or becoming ill and subjectively assessed involuntary exits, a desire to stay in work longer, do not necessarily coincide or overlap to a large extent [36]. In our study, the SA/DP group experiencing a downsizing did not display a decrease in purchases of psychotropic drugs of the same magnitude as the unexposed, further the decrease was somewhat delayed in comparison. In accordance with findings from a study on push factors in relation to retirement and subsequent psychosocial wellbeing [17], this could suggest that multiple push factors have a more negative impact on mental health than single or fewer factors. Another interpretation is that the unexposed, found to have a higher prevalence of purchases during the time before the exit, experienced a greater relief when they stopped working than the exposed group did. It is possible that the downsizing pushed people into sickness absence although being less severely ill compared to their unexposed counterparts. There is some evidence that when unemployment levels are high in economic downturns, a health selection on the labor market is taking place [37, 38]. The tolerance of suboptimal health is then reduced, pushing people out of work [35].

The unemployed exposed to downsizing, although they started at a lower prevalence of antidepressants, did not experience a decline in purchases of antidepressants before the exit, which the unexposed did. Like in the case of SA/DP, an exit via unemployment is likely to be perceived as involuntary regardless of whether it is in a context of downsizing or not. However, the lack of decline in purchases of antidepressant before exit among those exposed to downsizing/workplace closure could be a manifestation of adverse effects on mental health during the anticipation phase as indicated in another study on downsizing [22]. Improvements in mental health among the exposed might alternatively not take place during this phase to the same extent as for the unexposed because of poor working conditions at the organization undergoing changes [33, 34].

### Downsizing as a workplace push factor, the impact on purchases of psychotropic drugs

Previous studies on workplace factors as push factors for labor market exit has mainly focused on job satisfaction, changing job tasks, the demand-control model, and the effort-reward model [12, 16, 17, 39]. To our knowledge, only one study [18] has looked at labor market exit late in life in the context of downsizing, comparing early voluntary and involuntary old-age retirees to employees staying in work (voluntarily and involuntarily) after downsizing and their subsequent mental health. They concluded, that individuals in an involuntary state retired or still working, were worse off with regard to psychological wellbeing compared to those in a voluntary position. Generally, old-age retirees were better off compared to those still working, but mainly because they made a voluntary choice. Worst off were the involuntary retirees. Our study did not compare psychotropic drug purchases to those who did not exit after downsizing neither did we have direct information on voluntariness. However, some of our findings are in accordance with the study by Isaksson et al. (2000). Our finding that those exiting via old-age retirement or SA/DP experiencing downsizing, did not decrease purchases of psychotropic drugs around exit to the same extent as the unexposed, suggest that voluntariness matters. However, findings were not consistent in all exit groups and all types of drugs. What seem to matter most for prevalence of purchases of psychotropic drugs was the type of exit made. As pointed out by Halleröd et.al [15] both the type of exit made and the level of wellbeing after retirement can be regarded as outcomes of an accumulation of experiences throughout the life course. In addition, as concluded by Ebbinghaus and Radl (2015), voluntariness is best illustrated as a spectrum with different degrees of voluntariness [36], which further emphasize the complexity in studying the relationship between labor market exits late in life and mental health, acknowledging the role of voluntariness.

### Strengths and limitations

This large-scale study is based on the total working population in Sweden, represented by employees from both the private and public sectors. We used information from high quality registers with basically no missing data [40, 41]. Furthermore, by not using self-reported measures on exposure, nor on outcome, the results should not be affected by common method or recall bias. The long follow-up time made it possible for us to study changes in purchases during the anticipation phase, across the actual exit, and when adapting to the new situation. However, as information from the Swedish National Prescribed Drug Register was only available between 2005 and 2011, not all individuals have full information from 4 years before to 4 years after their exit. The findings, from early and late in the study period, are thereby based on fewer observations introducing a somewhat greater level of uncertainty across these years. In addition, purchases of psychotropic drugs have generally increased in Sweden over the study period. However, this general trend is unlikely to have influenced the findings as we adjusted our results for calendar time.

Furthermore, the inclusion of several types of psychotropic drugs can contribute to give a more complete picture regarding the potential associations on mental health with labor market exits around retirement age, with or without a downsizing event. Often, comorbidity exist between sleep problems, anxiety, and depression [42, 43], and sleep problems and anxiety can also act as a risk factor for depression [42, 44].

In the present study the Statistics on Dynamics of Enterprises and Establishments register (DEE) was used to classify employees as exposed or unexposed to downsizing. From the register we can identify workplace closures or staff reductions between two subsequent years. However, some misclassification may exist. If an employee works in a unit heavily affected by staff reductions but the workplace on average did not reduce their staffing with more than 18%, the individual was classified as unexposed and vice versa. Moreover, there may be other forms of organizational changes that were not measured in the study and may affect employees. This type of misclassification may have limited the possibility to observe any differences between exposed and unexposed.

To be registered in the prescribed drug register a person must first seek care, be diagnosed and prescribed the respective drug, and then redeem the drugs from the pharmacy. This means that some cases with poor mental health will not be detected. Hence, the prevalences presented in this study should not be interpreted as overall prevalences of poor mental health. Although the majority of purchases of psychotropic drugs are administered by Swedish pharmacies, drugs administered in e.g., hospitals or nursing homes are not captured [40]. Attitudes in favor of- and availability of drugs via the internet is also increasing [45]. Furthermore, we do not know if the individual actually used the drug. Information was available for several covariates of relevance, but there may be other potential confounders which were not measured that may contribute to systematic bias.

Regarding the generalizability of the finding from this study it is important to keep in mind that we studied people who were in paid work the year before their exit and the attempt was to study exits that were of a permanent type. Hence, the interrelationship between labor market exit, downsizing and pattern of purchases of psychotropic drugs may differ for people with very low labor market attachment and who exits and re-enter the labor market repeatedly. Furthermore, the study was conducted in Sweden, where many people work until they are 65 years old after which they are guaranteed at least a minimum pension, healthcare and medication are highly subsidized and paid out of pocket only up to certain level. Therefore, the findings may only be generalized to similar labor market and welfare contexts. In addition, in terms of generalizability, our sample is somewhat overrepresented by individuals around normal retirement age as we excluded all employees still in work by the end of the study period. However, we did adjust for age in all analysis and we did distinguish between different types of exits routes, via old-age pensions, long-term sickness absence, disability pension, or unemployment, which is important as these group are different in terms of health status, timing, resources and reasons for leaving their employment.

## Conclusion

The present study suggests that, although weakly, downsizing and workplace closure is associated with a higher level of purchases of psychotropic drugs around a labor market exit among those of retirement age. These finding thus supports the idea that level of voluntariness is an important aspect for labor market exits among older workers to have non-detrimental effects on mental health. Hence, the often applied strategy by employers, to first offer early retirement incentives to older employees during organizational downsizing should perhaps be reconsidered.

The findings in the present study, by using longitudinal data, including different labor exits and mental health outcomes, further contributes to a better understanding of the inconsistent findings about the influence of labor market exit and mental health in previous literature.

## Supplementary information

**Additional file 1.** Odds ratios for purchases of psychotropic drugs by exit group and type of drug, only adjusted for calendar time.

## Data Availability

The data used in this study is administered by the Division of Insurance Medicine, Karolinska Institutet, and cannot be made publically. According to the General Data Protection Regulation, the Swedish law SFS 2018:218, the Swedish Data Protection Act, the Swedish Ethical Review Act, and the Public Access to Information and Secrecy Act, these type of sensitive data can only be made available, after legal review, for researchers who meet the criteria for access to this type of sensitive and confidential data. Readers may contact Professor Kristina Alexanderson (kristina.alexanderson@ki.se) regarding the data. The data used in this study cannot be made publically available due to Swedish privacy regulations. Readers may contact Professor Kristina Alexanderson (kristina.alexanderson@ki.se) regarding IMAS data. Data from the Longitudinal integration database for health insurance and labor market studies (LISA) and the Dynamics on Enterprises and Establishments (DEE) can be directly requested from Statistics Sweden (microdata.individ@scb.se), and data from the Prescribed Drug Register can be requested from The National Board of Health and Welfare (registerservice@socialstyrelsen.se) in Sweden for researchers who meet the criteria for access to confidential data.

## Abbreviations

ATC: Anatomical therapeutic chemical DEE Dynamics on enterprises and establishments
GEE: Generalized estimating equations
IMAS: Insurance Medicine All Sweden
LISA: Longitudinal Integration Database for Health Insurance and Labor Market Studies
QIC: Quasi-likelihood under the independence model criterion
SA/DP: Sickness absence or disability pension
