# Supplementary material for: Labor market exit around retirement age in Sweden and trajectories of psychotropic drugs in a context of downsizing
Source: BMC Public Health. 2020 May 5;20:618. doi: 10.1186/s12889-020-08589-6 (PMC7201680; doi:10.1186/s12889-020-08589-6)
Supplement: Supplementary file 1 — Additional file 1. Odds ratios for purchases of psychotropic drugs by exit group and type of drug, only adjusted for calendar time. [file 12889_2020_8589_MOESM1_ESM.pdf]

	Pre exit	Peri exit	Post exit
	'-1 vs -4'	'+1 vs -1'	'+4 vs +1'
Old-age retirement			
Antidepressants			
Downsizing	0.90 (0.82-0.99)	0.92 (0.86-0.98)	0.94 (0.86-1.03)
No downsizing	0.90 (0.85-0.95)	0.88 (0.85-0.92)	0.92 (0.87-0.97)
Sedatives			
Downsizing	1.01 (0.93-1.10)	0.96 (0.91-1.01)	1.00 (0.93-1.08)
No downsizing	0.99 (0.94-1.04)	0.92 (0.89-0.95)	1.02 (0.98-1.07)
Anxiolytics			
Downsizing	1.04 (0.92-1.17)	0.93 (0.86-1.01)	0.97 (0.88-1.07)
No downsizing	0.95 (0.88-1.02)	0.97 (0.92-1.02)	0.97 (0.91-1.03)
SA/DP			
Antidepressants			
Downsizing	1.30 (1.18-1.45)	0.94 (0.88-1.00)	0.86 (0.78-0.95)
No downsizing	1.40 (1.30-1.50)	0.88 (0.84-0.92)	0.93 (0.87-1.00)
Sedatives			
Downsizing	1.23 (1.10-1.37)	1.01 (0.94-1.08)	1.02 (0.93-1.12)
No downsizing	1.27 (1.18–1.37)	0.92 (0.88-0.96)	0.99 (0.93-1.06)
Anxiolytics			
Downsizing	1.34 (1.17-1.53)	0.97 (0.90-1.06)	1.00 (0.89-1.11)
No downsizing	1.41 (1.28-1.55)	0.98 (0.92-1.04)	1.05 (0.97-1.13)
Unemployment			
Antidepressants			
Downsizing	1.13 (0.94-1.36)	1.02 (0.90-1.15)	0.93 (0.78-1.10)
No downsizing	0.88 (0.78-0.99)	0.88 (0.80-0.96)	0.80 (0.71-0.91)
Sedatives			
Downsizing	1.01 (0.85-1.21)	0.92 (0.82-1.04)	0.95 (0.81-1.11)
No downsizing	0.93 (0.82-1.06)	0.88 (0.81-0.96)	1.03 (0.91-1.15)
Anxiolytics			
Downsizing	0.92 (0.73-1.17)	1.02 (0.87-1.19)	1.10 (0.89-1.36)
No downsizing	1.01 (0.86-1.20)	0.94 (0.84-1.05)	0.92 (0.79-1.06)
